# The Role of Steroid Hormones in the Modulation of Neuroinflammation by Dietary Interventions

**DOI:** 10.3389/fendo.2016.00009

**Published:** 2016-02-04

**Authors:** Andrea Rodrigues Vasconcelos, João Victor Cabral-Costa, Caio Henrique Mazucanti, Cristoforo Scavone, Elisa Mitiko Kawamoto

**Affiliations:** ^1^Laboratory of Molecular Neuropharmacology, Department of Pharmacology, Institute of Biomedical Science, University of São Paulo, São Paulo, Brazil; ^2^Laboratory of Molecular and Functional Neurobiology, Department of Pharmacology, Institute of Biomedical Science, University of São Paulo, São Paulo, Brazil

**Keywords:** glucocorticoids, sex hormones, dietary energy restriction, high-fat diet, neuroinflammation

## Abstract

Steroid hormones, such as sex hormones and glucocorticoids, have been demonstrated to play a role in different cellular processes in the central nervous system, ranging from neurodevelopment to neurodegeneration. Environmental factors, such as calorie intake or fasting frequency, may also impact on such processes, indicating the importance of external factors in the development and preservation of a healthy brain. The hypothalamic–pituitary–adrenal axis and glucocorticoid activity play a role in neurodegenerative processes, including in disorders such as in Alzheimer’s and Parkinson’s diseases. Sex hormones have also been shown to modulate cognitive functioning. Inflammation is a common feature in neurodegenerative disorders, and sex hormones/glucocorticoids can act to regulate inflammatory processes. Intermittent fasting can protect the brain against cognitive decline that is induced by an inflammatory stimulus. On the other hand, obesity increases susceptibility to inflammation, while metabolic syndromes, such as diabetes, are associated with neurodegeneration. Consequently, given that gonadal and/or adrenal steroids may significantly impact the pathophysiology of neurodegeneration, *via* their effect on inflammatory processes, this review focuses on how environmental factors, such as calorie intake and intermittent fasting, acting through their modulation of steroid hormones, impact on inflammation that contributes to cognitive and neurodegenerative processes.

## Introduction

Obesity is now considered a worldwide epidemic, with up to 35% of adults being considered overweight or obese. Women are more likely to develop such a phenotype (1), with female rates of obesity in the United States rising from 31.5% of women aged 60 or older in 2003–2004 to 38.1% in 2011–2012 (2). Obesity is highly correlated with inflammation in many tissues, including the central nervous system (CNS) (3). Obesity and nutrient overload can trigger proinflammatory cytokines, such as tumor necrosis factor-alpha (TNF-α) and interleukin (IL)-1β, to build up in a number of affected tissues. Cells, including adipocytes and brain cells, respond to this metabolic stimulus through activation of different signaling pathways, including c-jun N-terminal kinase (JNK), inhibitor of nuclear factor kappa-B kinase (IKK), and the nuclear factor kappa-B (NF-κB) itself (3).

Diet plays a central role in obesity development, and a consensus on its exact influence is far from being defined. One way of seeing the effects of diet-related factors on obesity is evaluating leptin levels in the blood. Leptin resistance is a very common characteristic of obesity (4), and its serum levels are higher in obese humans when compared to healthy subjects (5). Regarding dietary intake, different factors affect leptin concentration and sensitivity differently, although several contradictory results can be found in the literature.

Regarding carbohydrate consumption, data are somewhat conflicting. While high glycemic index carbohydrate consumption or drastic high-carbohydrate diet (80% carbohydrate) may lead to leptin resistance (6, 7), different high-carbohydrate diets have no or the opposite effects on leptin sensitivity and blood concentrations (8, 9). On the other hand, fat intake has consistently been proven to be associated with a leptin resistance state (10, 11). There is, however, a divergence regarding the type of fat [saturated, mono, or polyunsaturated fatty acids (PUFA)] that is important for the effects on leptin levels (12, 13).

Accumulating data clearly show that a high-fat diet (HFD) negatively impacts on health, including increasing the incidence of cardiovascular diseases, diabetes, and overall mortality (14–16). HFD-exposed animals and humans also have an increased susceptibility to the development of a range of psychiatric disorders, which significantly correlates with body mass index (BMI) and obesity (17). Although BMI is widely used as an easy assessment of overall adiposity, studies show that abdominal fat deposition and visceral adiposity correlate more highly with metabolic (18) and psychiatric disorders (19). In that sense, another type of assessment, such as waist circumference and/or waist-to-hip ratio, is being used as a more relatable measurement that associates body fat and health disorders (18). Such correlation is greater in women than in men, which may indicate a difference between male and female responses to nutritional status in relation to mental health (20, 21).

On the other hand, dietary energy restriction (DER), achieved through a variety of protocols in which food intake is chronically or intermittently limited, can induce many beneficial outcomes, including *via* anti-inflammatory and antioxidant effects, that potentially increase lifespan [reviewed in Ref. (22)]. A wide body of data show food availability to affect both the activation and the rhythmicity of the hypothalamic–pituitary–adrenocortical (HPA) axis (23, 24). Furthermore, glucocorticoids, a group of steroid hormones, can modulate a plethora of processes in the organism, including immune function and energy metabolism (25–27). Therefore, the involvement of the HPA axis, especially of the glucocorticoid, rodent corticosterone (human cortisol), in the beneficial effects of DER has been extensively studied. Not only may DER modulate the HPA axis but it may also influence the hypothalamic–pituitary–gonadal (HPG) axis (28–32), thereby potentially interfering with the levels of sex hormones, such as androgens and estrogens.

The levels of both glucocorticoids and sex hormones seem to be strongly associated with inflammatory processes (26, 33–37). Therefore, this review focuses on assessing the role of these steroid hormones in neuroinflammation and the modulation exerted by dietary interventions such as HFD and DER on this process.

## Neuroinflammation and Steroidal Hormones

### Glucocorticoids

Many studies have shown that glucocorticoids exert anti-inflammatory effects in the organism (38). Glucocorticoids secreted by the adrenal glands after a physiological or psychogenic stressful stimulus promote anti-inflammatory and immunosuppressant actions through several genomic and non-genomic mechanisms, including the increase of anti-inflammatory gene expression (e.g., inhibitor of NF-κB, IκB-α, and IL-1 receptor antagonist) and the inhibition of NF-κB and of proinflammatory cytokines (e.g., TNF-α and IL-1β) (37, 39–41). These mitigating effects on inflammation are frequently exploited in clinical settings to treat a plethora of inflammatory and immune conditions (42, 43).

The synthesis of glucocorticoids and proinflammatory cytokines are interconnected *via* autoregulatory feedback loops, with corticosterone inhibiting the synthesis of proinflammatory cytokines and these cytokines stimulating the release of glucocorticoids through the upregulation of adrenocorticotropic hormone release from the pituitary gland (38, 44). Glucocorticoids levels are elevated after an inflammatory stimulus, including when induced by lipopolysaccharide (LPS) (45). Interestingly, disrupting the glucocorticoid signaling, by adrenalectomy or using glucocorticoid receptor (GR) antagonists, results in a much higher sensitivity of mice to the lethal effects of LPS (33, 46–51). As such, glucocorticoids are important for the resolution of inflammatory processes.

However, unlike the acute anti-inflammatory properties of, and protection afforded by, glucocorticoids, chronically elevated glucocorticoid levels are harmful, including in the CNS (52). Glucocorticoids can actually potentiate, rather than blunt, neuroinflammation (52–56). Over evolution, it is clear that stressful events may be accompanied by an immunological challenge, such as from tissue damage, with glucocorticoid-induced immune suppression, in such circumstances, being maladaptive (35).

Munhoz et al. (53) showed that elevated acute corticosterone levels are proinflammatory in the CNS, enhancing LPS-induced NF-κB activation and proinflammatory gene expression, which is prevented by GR antagonist treatment. In another study, high corticosterone levels induced by chronic stress increase the TNF-α release and microglia activation induced by intracortical LPS injection, in a GR-dependent manner (52). Importantly, in this study, this reactivation-enhancing effect of glucocorticoids occurred even when the chronic stress occurred after the inflammatory stimulus. Furthermore, various studies have demonstrated that glucocorticoids increase the vulnerability of neurons to numerous insults, such as excitotoxins and global ischemia, rising neuronal death and worsening neurological outcomes (54–56).

**Figure 1:**
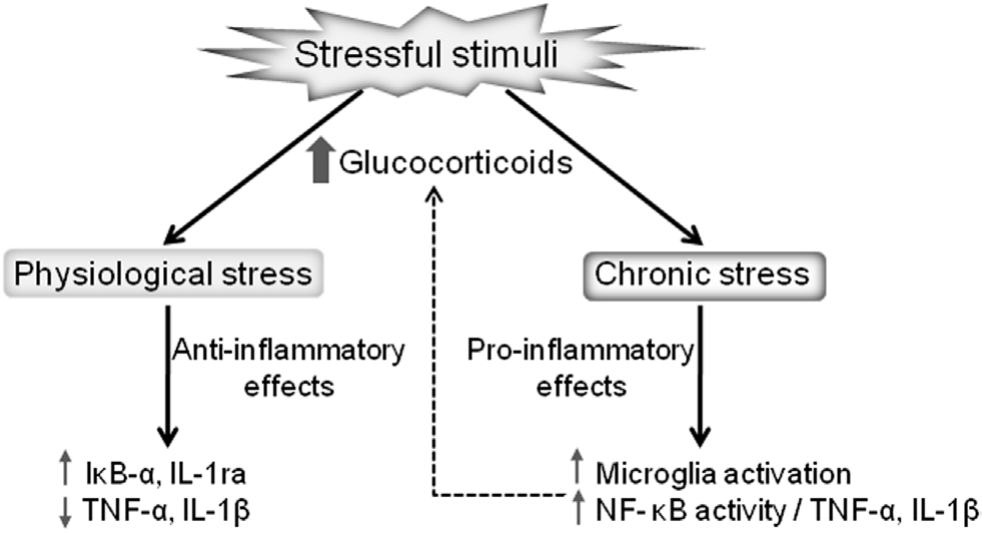
**Dual effects of stressful stimuli on glucocorticoid-mediated regulation of inflammation**. Glucocorticoids released after a mild stressor results in anti-inflammatory actions, reducing the proinflammatory cytokines production and increasing the expression of anti-inflammatory proteins, such as IL-1ra and IκB-α. On the other hand, pathological stressful stimuli lead to chronically elevated glucocorticoids promoting proinflammatory actions, including microglia activation and the consequent upregulation of the NF-κB proinflammatory cascade.

It is important to note that glucocorticoids can have differential impacts on brain immunity in different brain regions. For instance, the glucocorticoid-mediated potentiation of the inflammatory markers induced by LPS occurs in the frontal cortex and hippocampus but with the opposite effect being evident in the hypothalamus (57). Thus, glucocorticoids increase or decrease CNS inflammation depending on the dose, timing, duration of glucocorticoid exposure, and the type of glucocorticoid compound (35). The precise mechanism that contributes to these paradoxical responses, although of the utmost importance, is still unknown. What is currently known is that whether glucocorticoids exert anti- or proinflammatory effects is context-dependent, with variable response dependent upon concentration, time of exposure, the compound type, and the nature of the stimulus [reviewed in Ref. (35)]. The glucocorticoid-mediated effects on inflammation under physiological and chronic stress are summarized in Figure 1.

### Sex Hormones

The association of sex hormones and inflammation markers has been widely discussed in the literature. For instance, levels of testosterone and sex hormone-binding globulin (SHBG) were inversely correlated to markers of metabolic syndrome (58) and inflammation [e.g., C-reactive protein (59), γ-glutamyl transferase (60), and white blood cell and granulocyte count (36)]. Interestingly, male-derived cortical astrocytes show increased IL-6, TNF-α, and IL-1β mRNA levels in comparison to female-derived cells (61), indicating the importance of androgens as a modulator of central inflammation.

This correlation has also been supported by pharmacological approaches. Jayaraman et al. (62) observed that treatment with testosterone *in vitro* suppressed the increase in TNF-α expression in glial cultures from the cortices of animals submitted to a HFD. In addition, they observed a similar effect *in vivo* in gonadectomized mice, with a testosterone-mediated rescue of TNF-α and IL-1β mRNA levels in the cortex. Similarly, Khosla et al. (63) found an increase in serum TNF-α levels induced by acute, chemically induced hypogonadism in men. This effect was partially rescued by treatment with estrogen or testosterone, with a complete rescue being achieved by a combined therapy of both hormones (63). Accordingly, orchidectomized animals challenged with LPS show increased sickness behavior and increased levels of circulating IL-6 (64). This augmented reaction to LPS might be mediated by increased toll-like receptor (TLR)-4 signaling, as treatment with testosterone *in vitro* and *in vivo* decreases TLR-4 expression and sensitivity in macrophages (64). Testosterone also has a similar protective effect, in comparison with estradiol, in primary cultured neurons, where it prevents serum deprivation-induced apoptosis in an aromatase-independent manner, eliminating a possible indirect effect mediated by its conversion into estrogen (65).

In addition, testosterone is a potential neuroprotective factor against the inflammation associated with neurodegenerative disorders, such as Alzheimer’s disease (66) and multiple sclerosis (67). However, this literature is confounded by mixed results, with studies showing either no (60) or a positive correlation of testosterone levels and inflammation markers (68). As well as testosterone, estrogens have also been depicted as neuroprotective and/or anti-inflammatory agents. In microglia, estradiol has a very prominent inhibitory effect. Six hours after a subcutaneous injection of estradiol in ovariectomized (OVX) rats, LPS-induced macrophage activation is reduced by 60–90% in the cerebral cortex and hippocampus, as measured by reduced expression of ED-1/CD68. Similarly, in a mouse model of Alzheimer’s disease with plaque deposition, activated microglia surrounding amyloid plaques are greatly increased in OVX mice, which is reversed by an estradiol replacement therapy, although no effects over Aβ deposit levels were observed (69). Accordingly, an *in vitro* study showed similar estrogen effects, impairing microglial activation. The estrogens, estriol, and estradiol, as well as progesterone, suppress LPS-induced increase in inducible nitric oxide synthase (iNOS) expression and nitrite production in N9 microglial cells and in rat primary cultured microglia (70, 71). Estradiol was able to suppress nitric oxide (NO) and TNF-α increase, as well as cell death, in rat primary cultured glial cells challenged with LPS (72). Additionally, by stimulating BV-2 murine microglial cells with LPS, Baker et al. (73) showed estrogen pretreatment to decrease NO production, as well as iNOS and cyclooxygenase (COX)-2 expression, through interaction with the estrogen receptor (ER)-β (73). Albeit acute microglial activation could be essential to beneficial inflammatory processes, supporting the tissue in coping with stressors (74), the maintenance of a chronic inflammatory status is associated with the development of many pathologies, including neurodegenerative disorders such as Alzheimer’s disease. These studies point out to the importance of estrogens as regulators of microglial activity, thus highlighting the potential of this mechanism as a way to modulate dysregulated inflammation.

Estradiol also has anti-inflammatory effects in astrocytes. Treatment with either estradiol or ER modulators inhibits astrocyte proliferation to a plethora of insults, such as stab wound injury (75, 76), kainic acid ablation (77), and in a MPTP model of Parkinson’s disease (78). Additionally, estradiol also inhibits amyloid β (Aβ)-induced elevation in IL-1β and TNF-α levels, as well as COX-2 and iNOS expression, in primary cultured astrocytes (79). Estradiol treatment also attenuates the increased hippocampal expression of IL-1β and TNF-α, as well as astrocyte activation, in OVX rats, which is mediated *via* decreased NF-κB signaling (80). Moreover, a reduction in proinflammatory cytokine and chemokine mRNA content is observed in cultured astrocytes that have been prior-treated with estradiol and ER modulators before LPS stimulation (81).

Indeed, ER modulators seem to have anti-inflammatory effects, as proven in several *in vivo* and *in vitro* experimental models. Such data indicate that ER-α deletion, specifically in astrocytes (but not neurons), lowers the beneficial effects of an ER-α ligand treatment, in an experimental autoimmune encephalomyelitis (EAE) animal model, as seen by clinical function, central inflammation, and axonal loss on female mice (82). Additionally, astroglial cell death and mitochondrial function are rescued by an estradiol pretreatment in an *in vitro* oxygen/glucose deprivation model (83).

Likewise, *in vitro* astrocyte cultures have a diminished inflammatory response following ER manipulations, as seen by an ER-β-dependent reduction in NF-κB signaling (84), reduction in COX and iNOS expression (79), and overall reduction in proinflammatory cytokine release (84). In addition, estrogen has a positive effect on astrocytic glutamate re-uptake, by increasing glutamate transporters GLT-1 and GLAST (85), contributing to the functioning of glutamatergic synapses, as well as affording neuroprotection against the excitotoxic effects of raised levels of extracellular glutamate.

Many other *in vivo* models of inflammation also indicate estrogen to be neuroprotective. Estradiol reduces brain levels of TNF-α, IL-1β, and IL-6 in male rats in model of severe peripheral burning injury (34). Additionally, low doses of progesterone or high doses of estrogen attenuate the raised levels of IL-1β that are evident 24 h after a traumatic brain injury in female rats, although the sex hormones increased the cytokine levels in the early inflammatory phase (86). Interestingly, Zhang et al. (87) suggested that not only circulating but also brain-derived estrogen might act as anti-inflammatory modulator. They showed, in a model of global cerebral ischemia in female rats, that hippocampal astrocyte activation was associated with an increase in aromatase expression, with consequent elevations of estradiol levels in the CA1 region, while suppression of its expression through antisense oligonucleotides enhanced cell death as well as astrocyte and microglial activation (87).

Nonetheless, not all studies indicate beneficial effects of estrogens. Chronic estrogen treatment of OVX rats impairs their performance in a water maze test in a way that is similar to that of LPS. When both estrogen treatment and LPS were combined, performance was further decreased (88). Interestingly, although exerting an anti-inflammatory effect in young females, estradiol increases brain IL-1β levels in reproductively senescent rats (89), thus highlighting that aging might impair the protective response to estrogens. Accordingly, in a model of systemic lupus erythematosus in female mice, genetic deficiency of ER-α decreased Iba1^+^-activated microglia and rescued the cognitive deficit as assessed by the radial arm water maze test (90).

Overall, most studies suggest a protective effect of both testosterone and estrogens, although many factors may act to complicate such general conclusions, on occasion inverting these beneficial effects, which highlights the importance of further assessing the biological underpinnings that drive such complications. It is possible that the further assessment of testosterone/estrogen ratio – instead of single hormone level analyses – could help to further elucidate the reason for such paradoxical results, since it seems to have a greater physiological relevance (30). In addition, the complex effects of estrogens in inflammation might also be dependent on distinct activation of specific ER subtypes, ER-α and ER-β (84, 91, 92), and may also vary between different models.

## Involvement of Steroid Hormones in Dietary Interventions

### HFD and Glucocorticoids

Behavioral impacts of chronic exposure to a HFD can last for months, and their progression develops differently depending on which stage of the lifespan HFD exposure occurs. For example, rats chronically exposed to a lipid overload in their pre- and postnatal period exhibit anxiety-like behavior when adults, as measured by open field and elevated plus maze tasks (93, 94). HFD consumption during pregnancy impairs maternal behavior in mice, increasing circulating levels of corticosterone. Although the fetus is usually protected against maternal glucocorticoids due to placenta expression of 11beta-hydroxysteroid dehydrogenase type 2 (11β-HSD2), mice fed with a HFD have a lower expression of placental 11β-HSD2, rendering the fetus vulnerable to the effects of maternal circulating levels of glucocorticoids (95). Alterations in the gene expression of GRs in the amygdala as well as pro- and anti-inflammatory gene expression profiles in the amygdala and hippocampus of the offspring as adults could explain the increased anxiety-like behavior of animals exposed to HFD in pre- and perinatal periods of their early development (94, 96).

High-fat diet consumption by adult rats also alters their HPA axis response to stress, resulting in elevated glucocorticoid levels (97). Such altered response may be due to an increased noradrenergic input in the paraventricular nucleus (PVN) of the hypothalamus, an area responsible for controlling corticotrophin-releasing hormone (CRH) delivery to the pituitary. Indeed, HFD induces tyrosine hydroxylase expression in the PVN area and increases CRH levels in the median eminence, part of the hypophyseal portal system (98). Besides HPA axis regulation, HFD in adults has a clear impact on systemic and central inflammation, dysregulating inflammatory gene expression, which, among other disorders, plays a central role in the development of insulin resistance (99, 100).

Disruption of some components of corticosteroid signaling has also been reported in several rat brain regions following chronic HFD consumption. Regulation of mineralocorticoid receptors and GRs, respectively, seems to be region-specific, increasing in some brain areas and diminishing in others. After chronic exposure to HFD, hippocampi of female rats exhibit a lower expression of both corticosteroid receptors (101), while an increase in amygdala levels of MR and GR was observed (94). However, both of these alterations can have similar stress-regulatory effects, with amygdala GR activation enhancing the stress response, while a loss of the HPA axis suppressive effects of the hippocampal GR by HFD, leading to a loss of its inhibition of the stress response (102). As well as such hippocampal changes arising from chronic stress, they are also evident in wider neurodegenerative-associated processes, including aging (103, 104). In all these cases, reduced expression of hippocampal corticosteroid receptors is associated with an exaggerated response to stress, confirming the inhibitory effect of hippocampal projections on the HPA axis.

It is well known that NF-κB activity is affected by GR expression and signaling (35). Corticosterone, through GR activation, can induce apoptosis in lymphocytes (105), which explains the first observed data linking stress to immunosuppressant effects. Chronic supraphysiological levels of glucocorticoids are able to hinder immunity responses, reducing leukocyte count and impairing expression of several proinflammatory cytokines (106). Acting as an immunosuppressant, glucocorticoids have direct antagonizing effects upon NF-κB signaling, enhancing the expression of IκB and promoting the interaction between the GR and NF-κB in the nucleus, which, among many other effects, can decrease immune activation (106). On the other hand, increased immune response is also reported in initial phases of a stressful stimulus, an effect that is dependent upon basal levels of corticosterone (107). Given such a collection of data, it is not surprising that HFD can alter NF-κB activity and the expression of different inflammatory cytokines. In mouse peripheral tissues, the expression of NF-κB is increased in response to a chronic HFD. Enhanced NF-κB expression and activity was also reported in the amygdala of rats exposed to a HFD in their perinatal period (94), while diminished NF-κB expression is found in their hippocampus (101). Corroborating the central protective role of NF-κB, especially in the hippocampus, recent data show that a HFD can impair hippocampal neurogenesis, while elevating plasma corticosterone levels (108). Several other studies indicate different deleterious effects caused by a HFD upon hippocampal neuron functioning, including diminished BDNF production, impaired neuronal plasticity (109, 110), and working memory deficits (111). Interestingly, impaired hippocampal neurogenesis in HFD rodents does not correlate with fat accumulation but rather to serum corticosterone levels (108). In fact, high serum corticosterone levels affect proliferation, differentiation, and apoptosis in the dentate gyrus of rodents in different experimental settings (112, 113), indicating the significant role that alterations in the HPA axis play in HFD models.

The correlation between obesity and cognitive impairment has been extensively described in the past decade and several potential mechanisms were proposed trying to explain this link. Of these hypotheses, a few stand out. Impaired insulin and leptin signaling (114, 115) in the hippocampus and other memory-related brain regions is emerging as an interesting theory that forces us to further investigate the role of these two hormones in the physiology of the brain and specifically their purpose regarding synaptic function. However, considering the inflammatory pattern associated with obesity, it is not surprising that a large body of evidence supports the idea of a neuroinflammatory trigger to the cognitive deterioration associated with a HFD and obesity.

In this context, local proinflammatory cytokine production has been reported to occur in some brain regions after different HFD protocols (116–118). In all these cases, anti-inflammatory and/or antioxidant treatment (both pharmacological and non-pharmacological interventions) were able to revert HFD-induced cognitive impairment. This cognitive improvement seems to be accompanied not only by a reduction in inflammatory and oxidative stress markers but also with a reduced neuronal insulin resistance, driving a strong correlation between these factors.

Reinforcing the important role of neuroinflammation, several studies have also described an increase in blood–brain barrier (BBB) permeability caused by a HFD, in turn leaving the CNS more vulnerable to the inflammatory signals produced in peripheral tissues. Such increases in BBB permeability are associated with cognitive decrements (119–121). Interestingly, a few reports suggest that this HFD-induced BBB defect may lead not only to cognitive impairment but could also be the connection between Western diet consumption and Alzheimer’s disease (122, 123).

### HFD and Sex Hormones

The effects of dietary lipids upon sex hormones have been the focus of many studies since the early 1990s, particularly with regard to female sex hormones. Such interest in female hormones was triggered by data showing blood and urine estrogen levels to correlate positively with breast cancer risk in postmenopausal women (124). Coupled to the role of estrogens in the regulation of Alzheimer’s disease, manipulating estrogen levels has been extensively investigated, including how this can be achieved *via* dietary manipulations.

There is now a general consensus that a low-fat diet significantly reduces estrogen levels in healthy postmenopausal women (125, 126). Dietary effects on serum estradiol levels were assessed in a study comparing estradiol levels from Caucasian and Asian women (127). Postmenopausal Asian women consumed a total of 19% of their calories from lipids, whereas in Caucasian women, this percentage was 38%. Serum estradiol levels were significantly lower in Asian women, being 30–70% lower than postmenopausal Caucasian women. Dietary intervention studies in women to assess dietary lipid impact on estradiol levels usually apply a low-lipid diet, with only 10–25% of calories being lipid derived. Results from such studies show significant changes occurring as early as 3 months after the intervention, with the general serum estradiol reduction being around 13% (126, 128–130).

The consumption of omega-3 fatty acids is another possible dietary intervention that can modulate estradiol levels. Dietary omega-3 and omega-6 PUFA are both required in order to optimize health but can have differential effects on the inflammatory response. Omega-6 PUFA consumption increases the levels of a number of proinflammatory mediators, including prostaglandin E2 (PGE2) and leukotriene B4 (LB4), while omega-3 raises the levels of mediators with relatively lower inflammatory activity, such as prostaglandin E3 (PGE3) and leukotriene B5 (LB5). Both PGE2 and PGE3 and LB4 and LB5 are produced by the same enzymes, cyclooxygenase and 5-lipoxygenase, respectively. By enhancing substrate competition, omega-3 supplementation can reduce PGE2 and LB4 production, thereby attenuating the inflammatory response (131, 132). Furthermore, PGE2 can induce aromatase expression, thereby increasing the conversion of androgens to estrogens (133). This is an important mechanism that allows omega-3 PUFA consumption to inhibit estrogen production. Just as a low-lipid diet can decrease estradiol levels, a HFD can have the opposite effect. Young et al. (134) have shown a 36.6% increase in the plasma estradiol levels of postmenopausal women after 8 weeks of HFD, compared to that at baseline. However, this study was not able to show the influence of a low-fat, high omega-3 PUFA diet on estradiol levels.

While estradiol can have detrimental effects on peripheral tissues in postmenopausal women, including increasing breast cancer risk, studies in the CNS have shown some opposite effects. The protective role that sex hormones perform in the brain is well established, including from the use of several different experimental models, ranging from stroke to neurodegenerative disorders (135). Although the neuroprotective role of estrogen hormones is widely acknowledged, arising in part from data showing estrogens to increase anti-inflammatory mediators and protect against excitotoxicity, its use as a therapeutic agent is limited due to its activity in peripheral tissues, where it can lead to feminization and altered gonadal function as well as correlating positively with increased cancer risk, predominantly breast and endometrial cancers. Consequently, research has focused on the utility of using selective ER modulators. Tissue- and cell-specific ER modulators are a promising alternative, should they combine the desired pharmacological effect with little to no side effects.

Although not within the scope of this review, a HFD has opposite effects with regard to male sex hormone levels. Mice submitted to a HFD regimen of a duration of 10 weeks showed higher serum estradiol levels, as expected, but lower concentrations of both luteinizing, and consequently, testosterone hormones (136). Interestingly enough, metformin treatment of obese mice induced by HFD is capable of partially reverse obesity-induced elevated estradiol serum levels and decreased serum testosterone, while rescuing several fertility parameters (137).

### DER and Glucocorticoids

Dietary energy restriction can be psychologically stressful. Being characterized by food deprivation and starvation, it can be coupled to negative emotions, such as anxiety, depression, and irritability (138). As a stressful stimulus, DER can increase HPA axis activity in a variety of species, thereby increasing an important stress indicator, namely, circulating glucocorticoid hormone levels. Furthermore, the glucocorticoid increase during DER would be expected to modulate metabolic functions, including by enabling nutrient mobilization that may be further catabolized for energy, such as the stimulation of gluconeogenesis, protein catabolism, which increases the release of constituent amino acids, and lipolysis, which sensitizes adipose tissue stored as triglyceride to the action of lipolytic substances (growth hormone and catecholamines), resulting in glycerol and fatty acids (139).

The theory of hormesis, whereby mild stressors can be beneficial, may help to explain the DER mechanisms (140). Unlike other chronic stressful stimuli, DER can have many favorable effects for the organism, including counteracting inflammation, extending life span, and reducing the prevalence of age-related diseases. It is theorized that the DER potentiation of glucocorticoids release may contribute to increased stress resistance, protecting the organism not only against the stressor itself but also by upregulating adaptive pathways that protect the organism against the exacerbation of inflammation, infection, and metabolic disorders that can disturb homeostasis (141–144). Noteworthy, Dhurandhar et al. (145) recently proposed that the DER protective mechanisms may involve stress-related interceptive cues, as hunger in the absence of dietary restriction, as induced by a ghrelin agonist, promotes the same beneficial effects as DER, counteracting inflammation, aging, and neurodegeneration.

Among the mechanisms by which DER effects occur, neuroendocrinological alterations may play an important role (146). As previously noted, increased glucocorticoid concentrations following DER occur in various species. For instance, glucocorticoids are moderately increased by DER in rodents, where it is suggested to play a role in the DER effect (146–150). Free corticosterone levels are increased in rats after DER at any point in lifespan, when compared to age-matched *ad libitum* (151). This has led to the proposal that a lifetime DER-induced daily hyperadrenocorticism may retard aging (152). Levay et al. (24) tested different DER doses of calorie restriction (CR) in rats ranging from 12.5 to 50% and showed that all the doses caused an increase in corticosterone levels following a dose–response trend, with increasing restriction associated with higher glucocorticoid levels (24). Interestingly, a study that evaluated whether DER would similarly affect cortisol concentrations in wild mice not subjected to many generations of laboratory selection similarly showed that DER elevates corticosterone levels throughout life, suggesting that this DER effect is not altered by genetic breeding effects (153).

In humans, DER has been shown to increase perceived stress and circulating cortisol concentrations (154). Moreover, athletes have higher cortisol secretion following DER (155), as do individuals with anorexia nervosa (156). The study of eight participants who were subjected to DER for 2 years in a closed ecological space (Biosphere 2) also showed increased morning total cortisol (157). Interestingly, Grayson et al. (158) reported that rodent weight loss induced by DER increased basal HPA axis activity, unlike the same level of weight loss induced by bariatric surgery. Cortisol is usually released in a circadian rhythm, being an important aspect of the circadian system. Higher circulating cortisol levels are observed during the early morning, with lower levels evident around midnight. Remarkably, the elevated glucocorticoid levels after DER, which is accompanied by an oscillation in the levels of GRs, follow an altered circadian profile (147, 159). Another study, using 40% CR mice, in comparison to *ad libitum* fed controls, showed a 10-fold increase of plasma corticosteroid levels at 7:00 a.m., a 2-fold increase at 4:00 p.m., and no difference at 11:00 p.m. in the circadian cycle (160).

During Ramadan, adult Muslims refrain from eating during daytime. Ramadan can be considered a DER, in which the frequency of food consumption is restricted [intermittent fasting (IF)] but not the levels of calories consumed. Literature data suggest that Ramadan decreases the amplitude of the cortisol circadian rhythm by increasing its nocturnal levels while decreasing its diurnal circulating levels (161–163). A serum cortisol increase at 3:00 p.m. can occur in Ramadan (164). During Ramadan, although still within the normal reference ranges, immune cells can also significantly decrease, as well as proinflammatory cytokines and chemokines levels, with consequences, e.g., cancer-associated processes (165, 166).

However, although it has commonly been shown that DER increases cortisol levels in primates and rodents, there is also evidence to suggest otherwise. It seems that cortisol modulation by DER varies depending on the species, protocol, and other circumstances studied. For instance, it has recently been shown that a mild 25% CR diet does not alter the salivary cortisol levels of overweight men and women (167). In another study with obese individuals submitted to mild CR for 3 weeks, although reduced cortisol production and the metabolism of cortisol and cortisone were observed, there was no alteration on plasma cortisol levels. Conversely, in the same study, obese individuals starved for 6 days showed increased plasma cortisol levels (168). Also, Sticker et al. (169) showed that 50% CR in horses led to decreased plasma cortisol levels, compared to controls. Accordingly, Glade et al. (170) showed that during prolonged DER, young horses on an 80% CR also showed decreased cortisol levels, when compared with horses fed with meals containing 160% of their energy requirements.

Importantly, not in all glucocorticoid-sensitive cell types may derive stress inoculation benefits from DER-induced cortisol release. For instance, previous studies suggest that glucocorticoids can render hippocampal and cortical neurons more vulnerable to metabolic, excitotoxic, and oxidative damage (171). Although it was previously demonstrated that DER upregulates brain heat shock proteins (172, 173), GR activation can downregulate the expression of several genes, including heat shock protein 70, known to be important to counteract stress-induced cell damage (174). DER in rodents leads to glucocorticoid release that can reduce neuronal sensitivity to glucocorticoids by promoting a feedback suppression on the levels of the potentially damaging GR, thereby increasing the resistance to injury (175).

A large body of evidence strengthens the proposed link between glucocorticoids and the anti-inflammatory effects of DER in rodents. Besides its metabolic actions, glucocorticoids are also known for their anti-inflammatory activities due to the inhibition of key inflammatory transcriptional regulators, such as the activator protein-1 (AP-1) and NF-κB, known to activate the transcription of proinflammatory cytokines (39).

Dietary energy restriction has also been linked to protection against inflammation. Vasconcelos et al. (176) showed that the IF protocol prevents or mitigates cognitive deficits, inflammatory genes transcription, and the diverse array of systemic LPS-induced cytokines in the brain and the periphery. Furthermore, 50% CR also reduces sickness behavior, fever, and peripheral immune markers following LPS injection, which was also modulated by DER duration (177, 178). In this DER protocol, elevated circulating corticosterone levels were evident, which the authors suggest contributes to the diminished proinflammatory signals in these animals (178). In another study, 40% CR in mice also prevented the LPS-induced proinflammatory cytokines upregulation, which was again accompanied by increased glucocorticoid production (179). Supporting this theory, previous data reported that adrenalectomized mice are much more sensitive to the lethal effect of LPS, which is prevented by the pretreatment with dexamethasone (33, 49). Such data highlight the immune-regulatory effects of glucocorticoids, including in CR paradigms.

Inflammation has long been associated with the development of cancer, which partly explains the association of obesity and tumorigenesis. By contrast, DER powerfully inhibits the development of cancer in many studies (180), including in wild mice as well as laboratory-reared rodents, indicating that laboratory selection does not interfere in this effect (153). Both topical and oral glucocorticoid treatments decrease tumor development in rodents (181–185). Adrenalectomy reverses the inhibition of tumorigenesis by DER in mice and glucocorticoids supplementation restores it, suggesting that adrenal hormones play an important role in mediating this DER effect (186–188).

An extensive literature shows inflammation to be a risk factor for cognitive impairment and dementia, with neuroinflammatory processes contributing to neurodegeneration (189, 190). Most neurodegenerative conditions are associated with chronic inflammation, which is widely accepted as contributing to the pathophysiology of neurodegenerative conditions (191–193). DER can prevent or attenuate inflammation associated with neurodegeneration (194–196). Studies also suggest that glucocorticoids play an important role, with some differential effects that are dependent on whether they are applied acutely or chronically, in neurodegeneration, such as in Alzheimer’s disease, being associated with chronic glucocorticoids (197–199), while acute conditions, such as ischemic stroke, benefiting from the acute effects of glucocorticoids (171). Such effects are relevant both clinically and in animal models.

Despite the DER-induced increase in glucocorticoids classically known for their anti-inflammatory action, studies have shown mixed results when evaluating the immune response to infection and pathogen clearance efficiency in animals submitted to DER protocols. In primates and rodent studies, while DER can improve clearance and survival after bacterial infection and enhance interferon gamma (IFN-γ) production, it can promote a deficient innate immune response with reduced ability to control infections by monocytes and macrophages (200–204). Patel and Finch (205) suggest that DER-induced glucocorticoid release may promote a differential effect on immunity in different parts of the organism, for instance, activating pathways close to the infectious focus that are not suppressed by glucocorticoids and attenuating inflammation at other locations.

**Figure 2:**
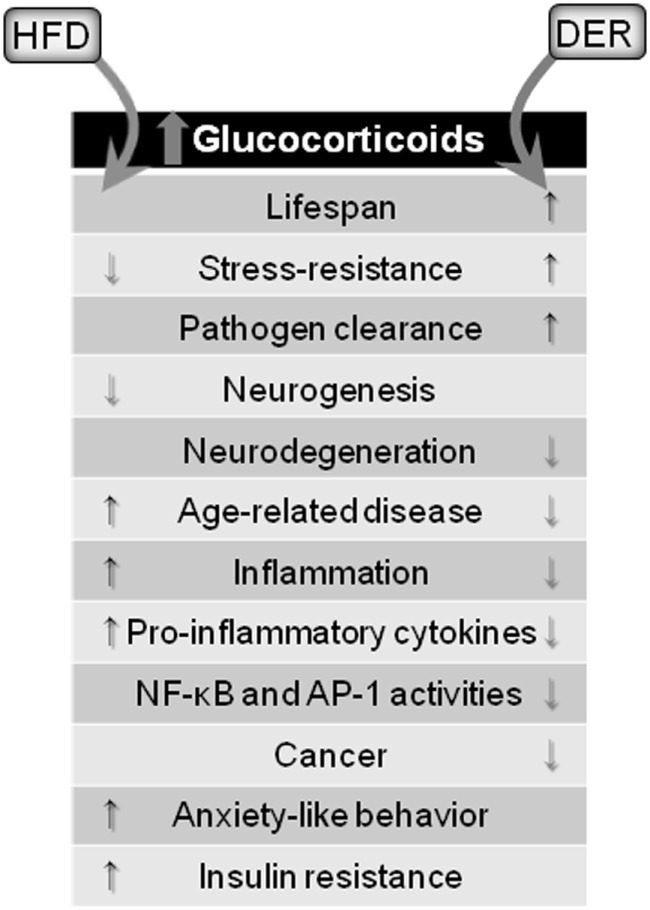
**Glucocorticoid-mediated effects of dietary interventions**. Both HFD and DER results in increased blood concentrations of glucocorticoids. However, opposing effects are observed. HFD, *via* glucocorticoids release, cause detrimental effects to the brain and organism, while DER-induced glucocorticoids release leads to protective effects.

A brief summary of DER and HFD modulatory effects on neuroinflammation through glucocorticoid signaling is outlined in Figure 2.

### DER and Sex Hormones

Numerous studies have demonstrated that DER protocols affect sex hormone levels. In rhesus monkeys, for instance, 30% CR deferred the age-related dehydroepiandrosterone decline in males (28). Accordingly, Levay et al. (24) observed a dose–response decrease in testosterone levels in male rats with increasing CR severity. This effect was also observed for the IF protocol (32). A severe CR protocol in men also decreased levels of testosterone and estradiol while increasing levels of SHBG (31). In women, IF also increases SHBG levels, although CR more significantly reduces dehydroepiandrosterone levels (206). In the Biosphere 2 study, Walford et al. (157) observed an increase in levels of androstenedione and SHBG in humans (both in male and female participants), while the levels of estradiol, but not testosterone, decreased in men.

In contrast to these results, Martin et al. (29) observed that male rats submitted to IF or females under 40% CR had increased levels of testosterone. In a subsequent study, they observed that either a 40% CR or an IF protocol increased the testosterone/estrogen ratio, consistent with a hyper-masculinization state. A similar effect was also observed in females under the 40% CR protocol (30). Furthermore, Kumar and Kaur (32) showed that IF induced a significant decrease in luteinizing hormone, associated with diminished levels of estradiol in female rats, which completely suppressed the estrous cycle. Other studies also showed DER to inhibit estradiol levels (29, 30).

**Figure 3:**
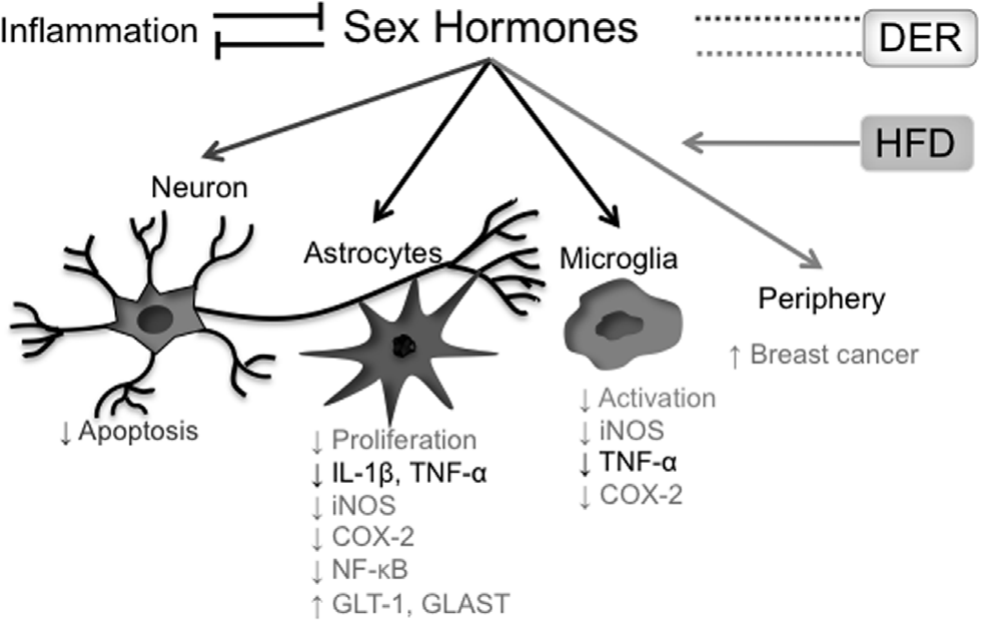
**Effects of sex hormones on the inflammatory process**. Presence of systemic inflammatory markers correlates inversely with blood concentrations of sex hormones, while hormonal reposition reduces both central and peripheral cytokine production. In the CNS, testosterone has protective roles both in neurons and glial cells, where it shows an anti-inflammatory action. Estradiol also has anti-inflammatory properties in glial cells (astrocytes and microglia). On the other hand, estradiol levels in postmenopausal women positively correlate with breast cancer incidence. A HFD raises estradiol blood levels in postmenopausal women, increasing breast cancer risk. The effects of different DER protocols on sex hormone levels are more controversial. In black, effects of and on both testosterone and estradiol; in light gray, effects related to estradiol; in dark gray, effects related to testosterone.

In conclusion, the effects of both DER interventions on sex hormones levels – including the testosterone/estrogen ratio – as well as the relevance of this modulation on DER anti-inflammatory properties are still obscure and have yet to be clarified. The interrelationship between sex hormones and neuroinflammation, as discussed above, is summarized in Figure 3.

## Concluding Remarks

In conclusion, glucocorticoids have been historically characterized as mediators of many anti-inflammatory effects observed within DER protocols, closely implicating glucocorticoid pathways in DER, including in the development of future pharmacological interventions that could mimic DER benefits. In contrast, extensive data support the hypothesis that the detrimental effects of a HFD upon cognitive function and behavior are caused by enhanced glucocorticoid signaling accompanied by neuroinflammation. As such, it is clear that there is more to glucocorticoid effects than simply its serum levels. Although both DER and HFD contribute to enhanced glucocorticoid blood concentration, its effects are quite opposite regarding health and, specifically, inflammation. Also, DER may induce its positive effects through other different mechanisms not related to glucocorticoid signaling, as may be the case for the detrimental effects of a HFD.

Given that androgen and estrogen levels also appear to be variably modulated by DER interventions and overall dietary lipid load, which is at least partly dependent on sex, age, and inflammatory status, it is possible that these hormones could have a relevant role to play in DER anti-inflammatory mechanisms and HFD-induced inflammation. However, this effect is still unclear. It is therefore important that future research should better clarify the role that such sex hormones play in DER and HFD mechanisms.

## Author Contributions

All the authors contributed to the design of the paper, literature review, writing of the manuscript, and creation of the figures.

## Conflict of Interest Statement

The authors declare that the research was conducted in the absence of any commercial or financial relationships that could be construed as a potential conflict of interest.

## Abbreviations

11β-HSD2, 11beta-hydroxysteroid dehydrogenase type 2; AP-1, activator protein-1; Aβ, amyloid β; BBB, blood–brain barrier; BMI, body mass index; CNS, central nervous system; COX, cyclooxygenase; CR, calorie restriction; CRH, corticotrophin-releasing hormone; DER, dietary energy restriction; EAE, experimental autoimmune encephalomyelitis; ER, estrogen receptor; GR, glucocorticoid receptor; HFD, high-fat diet; HPA, hypothalamic–pituitary–adrenocortical; HPG, hypothalamic–pituitary–gonadal; IκB-α, inhibitor of nuclear factor kappa-B; IF, intermittent fasting; IFN-γ, interferon gamma; IKK, inhibitor of nuclear factor kappa-B kinase; IL, interleukin; iNOS, inducible nitric oxide synthase; JNK, c-jun N-terminal kinase; LB4, leukotriene B4; LB5, leukotriene B5; LPS, lypopolysaccharide; MR, mineralocorticoid receptor; NF-κB, nuclear factor kappa-B; NO, nitric oxide; OVX, ovariectomized; PGE2, prostaglandin E2; PGE3, prostaglandin E3; PUFA, polyunsaturated fatty acids; PVN, paraventricular nucleus; SHBG, sex hormone-binding globulin; TLR, toll-like receptor; TNF-α, tumor necrosis factor-alpha.

## References

[1] Mitchell S Shaw D. The worldwide epidemic of female obesity. Best Pract Res Clin Obstet Gynaecol (2015) 29(3):289–99. 10.1016/j.bpobgyn.2014.10.002 25487257

[2] Ogden CL Carroll MD Kit BK Flegal KM. Prevalence of childhood and adult obesity in the United States, 2011-2012. JAMA (2014) 311(8):806–14. 10.1001/jama.2014.732 24570244 PMC4770258

[3] Gregor MF Hotamisligil GS Inflammatory mechanisms in obesity. Annu Rev Immunol (2011) 29:415–45. 10.1146/annurev-immunol-031210-101322 21219177

[4] Caro JF Sinha MK Kolaczynski JW Zhang PL Considine RV Leptin: the tale of an obesity gene. Diabetes (1996) 45(11):1455–62. 10.2337/diab.45.11.1455 8866547

[5] Maffei M Halaas J Ravussin E Pratley RE Lee GH Zhang Y Leptin levels in human and rodent: measurement of plasma leptin and ob RNA in obese and weight-reduced subjects. Nat Med (1995) 1(11):1155–61. 10.1038/nm1195-1155 7584987

[6] Herrmann TS Bean ML Black TM Wang P Coleman RA. High glycemic index carbohydrate diet alters the diurnal rhythm of leptin but not insulin concentrations. Exp Biol Med (Maywood) (2001) 226(11):1037–44. 11743140 10.1177/153537020122601111

[7] Lopes IM Forga L Martinez JA. Effects of leptin resistance on acute fuel metabolism after a high carbohydrate load in lean and overweight young men. J Am Coll Nutr (2001) 20(6):643–8. 10.1080/07315724.2001.10719162 11771681

[8] Weigle DS Cummings DE Newby PD Breen PA Frayo RS Matthys CC Roles of leptin and ghrelin in the loss of body weight caused by a low fat, high carbohydrate diet. J Clin Endocrinol Metab (2003) 88(4):1577–86. 10.1210/jc.2002-021262 12679442

[9] Kong A Neuhouser ML Xiao L Ulrich CM McTiernan A Foster-Schubert KE. Higher habitual intake of dietary fat and carbohydrates are associated with lower leptin and higher ghrelin concentrations in overweight and obese postmenopausal women with elevated insulin levels. Nutr Res (2009) 29(11):768–76. 10.1016/j.nutres.2009.10.013 19932865 PMC2784426

[10] Martin LJ Siliart B Lutz TA Biourge V Nguyen P Dumon HJ. Postprandial response of plasma insulin, amylin and acylated ghrelin to various test meals in lean and obese cats. Br J Nutr (2010) 103(11):1610–9. 10.1017/S000711450999359X 20100379

[11] Roberts CK Berger JJ Barnard RJ. Long-term effects of diet on leptin, energy intake, and activity in a model of diet-induced obesity. J Appl Physiol (1985) (2002) 93(3):887–93. 10.1152/japplphysiol.00224.2002 12183482

[12] Hynes GR Heshka J Chadee K Jones PJ. Effects of dietary fat type and energy restriction on adipose tissue fatty acid composition and leptin production in rats. J Lipid Res (2003) 44(5):893–901. 10.1194/jlr.M200318-JLR200 12562868

[13] Higuchi T Shirai N Saito M Suzuki H Kagawa Y. Levels of plasma insulin, leptin and adiponectin, and activities of key enzymes in carbohydrate metabolism in skeletal muscle and liver in fasted ICR mice fed dietary n-3 polyunsaturated fatty acids. J Nutr Biochem (2008) 19(9):577–86. 10.1016/j.jnutbio.2007.08.001 17911005

[14] Hill JO Peters JC. Environmental contributions to the obesity epidemic. Science (1998) 280(5368):1371–4. 10.1126/science.280.5368.1371 9603719

[15] Flegal KM Graubard BI Williamson DF Gail MH. Excess deaths associated with underweight, overweight, and obesity. JAMA (2005) 293(15):1861–7. 10.1001/jama.293.15.1861 15840860

[16] Kahn SE Hull RL Utzschneider KM. Mechanisms linking obesity to insulin resistance and type 2 diabetes. Nature (2006) 444(7121):840–6. 10.1038/nature05482 17167471

[17] Carpiniello B Pinna F Pillai G Nonnoi V Pisano E Corrias S Obesity and psychopathology. A study of psychiatric comorbidity among patients attending a specialist obesity unit. Epidemiol Psichiatr Soc (2009) 18(2):119–27. 19526743

[18] Lim U Ernst T Wilkens LR Albright CL Lum-Jones A Seifried A Susceptibility variants for waist size in relation to abdominal, visceral, and hepatic adiposity in postmenopausal women. J Acad Nutr Diet (2012) 112(7):1048–55. 10.1016/j.jand.2012.03.034 22889634 PMC3419359

[19] Zhi T Wang Q Liu Z Zhu Y Wang Y Shi R Body mass index, waist circumference and waist-hip ratio are associated with depressive symptoms in older Chinese women: results from the Rugao Longevity and Ageing Study (RuLAS). Aging Ment Health (2015) 21:1–6. 10.1080/13607863.2015.1124837 26689763

[20] Desai RA Manley M Desai MM Potenza MN. Gender differences in the association between body mass index and psychopathology. CNS Spectr (2009) 14(7):372–83. 19773713 10.1017/s1092852900023026 PMC3708686

[21] McCrea RL Berger YG King MB. Body mass index and common mental disorders: exploring the shape of the association and its moderation by age, gender and education. Int J Obes (Lond) (2012) 36(3):414–21. 10.1038/ijo.2011.65 21427699

[22] Longo VD Mattson MP. Fasting: molecular mechanisms and clinical applications. Cell Metab (2014) 19(2):181–92. 10.1016/j.cmet.2013.12.008 24440038 PMC3946160

[23] Garcia-Belenguer S Oliver C Mormede P. Facilitation and feedback in the hypothalamo-pituitary-adrenal axis during food restriction in rats. J Neuroendocrinol (1993) 5(6):663–8. 10.1111/j.1365-2826.1993.tb00537.x 8680439

[24] Levay EA Tammer AH Penman J Kent S Paolini AG. Calorie restriction at increasing levels leads to augmented concentrations of corticosterone and decreasing concentrations of testosterone in rats. Nutr Res (2010) 30(5):366–73. 10.1016/j.nutres.2010.05.001 20579529

[25] McDowell GH Hormonal control of glucose homoeostasis in ruminants. Proc Nutr Soc (1983) 42(2):149–67. 10.1079/PNS19830021 6351077

[26] Wilckens T De Rijk R. Glucocorticoids and immune function: unknown dimensions and new frontiers. Immunol Today (1997) 18(9):418–24. 10.1016/S0167-5699(97)01111-0 9293156

[27] Kelly JJ Mangos G Williamson PM Whitworth JA Cortisol and hypertension. Clin Exp Pharmacol Physiol (1998) 25:S51–6. 10.1111/j.1440-1681.1998.tb02301.x 9809193

[28] Mattison JA Lane MA Roth GS Ingram DK. Calorie restriction in rhesus monkeys. Exp Gerontol (2003) 38(1–2):35–46. 10.1016/S0531-5565(02)00146-8 12543259

[29] Martin B Pearson M Kebejian L Golden E Keselman A Bender M Sex-dependent metabolic, neuroendocrine, and cognitive responses to dietary energy restriction and excess. Endocrinology (2007) 148(9):4318–33. 10.1210/en.2007-0161 17569758 PMC2622430

[30] Martin B Pearson M Brenneman R Golden E Wood W Prabhu V Gonadal transcriptome alterations in response to dietary energy intake: sensing the reproductive environment. PLoS One (2009) 4(1):e4146. 10.1371/journal.pone.0004146 19127293 PMC2607546

[31] Cangemi R Friedmann AJ Holloszy JO Fontana L. Long-term effects of calorie restriction on serum sex-hormone concentrations in men. Aging Cell (2010) 9(2):236–42. 10.1111/j.1474-9726.2010.00553.x 20096034 PMC3569090

[32] Kumar S Kaur G. Intermittent fasting dietary restriction regimen negatively influences reproduction in young rats: a study of hypothalamo-hypophysial-gonadal axis. PLoS One (2013) 8(1):e52416. 10.1371/journal.pone.0052416 23382817 PMC3558496

[33] Parant M Le Contel C Parant F Chedid L. Influence of endogenous glucocorticoid on endotoxin-induced production of circulating TNF-alpha. Lymphokine Cytokine Res (1991) 10(4):265–71. 1932370

[34] Gatson JW Maass DL Simpkins JW Idris AH Minei JP Wigginton JG. Estrogen treatment following severe burn injury reduces brain inflammation and apoptotic signaling. J Neuroinflammation (2009) 6:30. 10.1186/1742-2094-6-30 19849845 PMC2774304

[35] Sorrells SF Caso JR Munhoz CD Sapolsky RM. The stressed CNS: when glucocorticoids aggravate inflammation. Neuron (2009) 64(1):33–9. 10.1016/j.neuron.2009.09.032 19840546 PMC4782919

[36] Brand JS van der Schouw YT Dowsett M Folkerd E Luben RN Wareham NJ Testosterone, SHBG and differential white blood cell count in middle-aged and older men. Maturitas (2012) 71(3):274–8. 10.1016/j.maturitas.2011.12.007 22221653

[37] Bellavance MA Rivest S. The HPA – immune axis and the immunomodulatory actions of glucocorticoids in the brain. Front Immunol (2014) 5:136. 10.3389/fimmu.2014.00136 24744759 PMC3978367

[38] Homo-Delarche F Fitzpatrick F Christeff N Nunez EA Bach JF Dardenne M. Sex steroids, glucocorticoids, stress and autoimmunity. J Steroid Biochem Mol Biol (1991) 40(4–6):619–37. 10.1016/0960-0760(91)90285-D 1958562

[39] De Bosscher K Vanden Berghe W Haegeman G. Mechanisms of anti-inflammatory action and of immunosuppression by glucocorticoids: negative interference of activated glucocorticoid receptor with transcription factors. J Neuroimmunol (2000) 109(1):16–22. 10.1016/S0165-5728(00)00297-6 10969176

[40] Almawi WY Melemedjian OK. Negative regulation of nuclear factor-kappaB activation and function by glucocorticoids. J Mol Endocrinol (2002) 28(2):69–78. 10.1677/jme.0.0280069 11932204

[41] Franchimont D. Overview of the actions of glucocorticoids on the immune response: a good model to characterize new pathways of immunosuppression for new treatment strategies. Ann N Y Acad Sci (2004) 1024:124–37. 10.1196/annals.1321.009 15265777

[42] Kadmiel M Cidlowski JA. Glucocorticoid receptor signaling in health and disease. Trends Pharmacol Sci (2013) 34(9):518–30. 10.1016/j.tips.2013.07.003 23953592 PMC3951203

[43] Vandevyver S Dejager L Tuckermann J Libert C. New insights into the anti-inflammatory mechanisms of glucocorticoids: an emerging role for glucocorticoid-receptor-mediated transactivation. Endocrinology (2013) 154(3):993–1007. 10.1210/en.2012-2045 23384835

[44] Besedovsky HO del Rey A Klusman I Furukawa H Monge Arditi G Kabiersch A. Cytokines as modulators of the hypothalamus-pituitary-adrenal axis. J Steroid Biochem Mol Biol (1991) 40(4–6):613–8. 10.1016/0960-0760(91)90284-C 1659887

[45] Hansen MK Nguyen KT Fleshner M Goehler LE Gaykema RP Maier SF Effects of vagotomy on serum endotoxin, cytokines, and corticosterone after intraperitoneal lipopolysaccharide. Am J Physiol Regul Integr Comp Physiol (2000) 278(2):R331–6. 10666132 10.1152/ajpregu.2000.278.2.R331

[46] Bertini R Bianchi M Ghezzi P. Adrenalectomy sensitizes mice to the lethal effects of interleukin 1 and tumor necrosis factor. J Exp Med (1988) 167(5):1708–12. 10.1084/jem.167.5.1708 3259257 PMC2188949

[47] MacPhee IA Antoni FA Mason DW. Spontaneous recovery of rats from experimental allergic encephalomyelitis is dependent on regulation of the immune system by endogenous adrenal corticosteroids. J Exp Med (1989) 169(2):431–45. 10.1084/jem.169.2.431 2783450 PMC2189214

[48] Edwards CK III Yunger LM Lorence RM Dantzer R Kelley KW. The pituitary gland is required for protection against lethal effects of *Salmonella typhimurium*. Proc Natl Acad Sci U S A (1991) 88(6):2274–7. 10.1073/pnas.88.6.2274 1900940 PMC51213

[49] Ramachandra RN Sehon AH Berczi I. Neuro-hormonal host defence in endotoxin shock. Brain Behav Immun (1992) 6(2):157–69. 10.1016/0889-1591(92)90015-G 1504369

[50] Ruzek MC Pearce BD Miller AH Biron CA. Endogenous glucocorticoids protect against cytokine-mediated lethality during viral infection. J Immunol (1999) 162(6):3527–33. 10092810

[51] Nadeau S Rivest S. Glucocorticoids play a fundamental role in protecting the brain during innate immune response. J Neurosci (2003) 23(13):5536–44. 12843254 10.1523/JNEUROSCI.23-13-05536.2003 PMC6741270

[52] de Pablos RM Villaran RF Arguelles S Herrera AJ Venero JL Ayala A Stress increases vulnerability to inflammation in the rat prefrontal cortex. J Neurosci (2006) 26(21):5709–19. 10.1523/JNEUROSCI.0802-06.2006 16723527 PMC6675274

[53] Munhoz CD Sorrells SF Caso JR Scavone C Sapolsky RM. Glucocorticoids exacerbate lipopolysaccharide-induced signaling in the frontal cortex and hippocampus in a dose-dependent manner. J Neurosci (2010) 30(41):13690–8. 10.1523/JNEUROSCI.0303-09.2010 20943909 PMC3842494

[54] Sapolsky RM Pulsinelli WA. Glucocorticoids potentiate ischemic injury to neurons: therapeutic implications. Science (1985) 229(4720):1397–400. 10.1126/science.4035356 4035356

[55] Dinkel K MacPherson A Sapolsky RM. Novel glucocorticoid effects on acute inflammation in the CNS. J Neurochem (2003) 84(4):705–16. 10.1046/j.1471-4159.2003.01604.x 12562515

[56] Sorrells SF Munhoz CD Manley NC Yen S Sapolsky RM. Glucocorticoids increase excitotoxic injury and inflammation in the hippocampus of adult male rats. Neuroendocrinology (2014) 100(2–3):129–40. 10.1159/000367849 25228100 PMC4304880

[57] Munhoz CD Lepsch LB Kawamoto EM Malta MB Lima Lde S Avellar MC Chronic unpredictable stress exacerbates lipopolysaccharide-induced activation of nuclear factor-kappaB in the frontal cortex and hippocampus via glucocorticoid secretion. J Neurosci (2006) 26(14):3813–20. 10.1523/JNEUROSCI.4398-05.2006 16597735 PMC6674142

[58] Laaksonen DE Niskanen L Punnonen K Nyyssonen K Tuomainen TP Salonen R Sex hormones, inflammation and the metabolic syndrome: a population-based study. Eur J Endocrinol (2003) 149(6):601–8. 10.1530/eje.0.1490601 14641004

[59] Kupelian V Chiu GR Araujo AB Williams RE Clark RV McKinlay JB. Association of sex hormones and C-reactive protein levels in men. Clin Endocrinol (Oxf) (2010) 72(4):527–33. 10.1111/j.1365-2265.2009.03713.x 19769617 PMC2866020

[60] Haring R Baumeister SE Volzke H Dorr M Kocher T Nauck M Prospective inverse associations of sex hormone concentrations in men with biomarkers of inflammation and oxidative stress. J Androl (2012) 33(5):944–50. 10.2164/jandrol.111.015065 22207707

[61] Santos-Galindo M Acaz-Fonseca E Bellini MJ Garcia-Segura LM. Sex differences in the inflammatory response of primary astrocytes to lipopolysaccharide. Biol Sex Differ (2011) 2:7. 10.1186/2042-6410-2-7 21745355 PMC3143074

[62] Jayaraman A Lent-Schochet D Pike CJ. Diet-induced obesity and low testosterone increase neuroinflammation and impair neural function. J Neuroinflammation (2014) 11:162. 10.1186/s12974-014-0162-y 25224590 PMC4190446

[63] Khosla S Atkinson EJ Dunstan CR O’Fallon WM. Effect of estrogen versus testosterone on circulating osteoprotegerin and other cytokine levels in normal elderly men. J Clin Endocrinol Metab (2002) 87(4):1550–4. 10.1210/jcem.87.4.8397 11932280

[64] Rettew JA Huet-Hudson YM Marriott I. Testosterone reduces macrophage expression in the mouse of toll-like receptor 4, a trigger for inflammation and innate immunity. Biol Reprod (2008) 78(3):432–7. 10.1095/biolreprod.107.063545 18003947

[65] Hammond J Le Q Goodyer C Gelfand M Trifiro M LeBlanc A. Testosterone-mediated neuroprotection through the androgen receptor in human primary neurons. J Neurochem (2001) 77(5):1319–26. 10.1046/j.1471-4159.2001.00345.x 11389183

[66] Butchart J Birch B Bassily R Wolfe L Holmes C. Male sex hormones and systemic inflammation in Alzheimer disease. Alzheimer Dis Assoc Disord (2013) 27(2):153–6. 10.1097/WAD.0b013e318258cd63 22596080

[67] Spence RD Voskuhl RR. Neuroprotective effects of estrogens and androgens in CNS inflammation and neurodegeneration. Front Neuroendocrinol (2012) 33(1):105–15. 10.1016/j.yfrne.2011.12.001 22209870 PMC3616506

[68] Filgueira FP Lobato NS DosSantos RA Oliveira MA Akamine EH Tostes RC Endogenous testosterone increases leukocyte-endothelial cell interaction in spontaneously hypertensive rats. Life Sci (2012) 90(17–18):689–94. 10.1016/j.lfs.2012.03.009 22498877

[69] Vegeto E Belcredito S Ghisletti S Meda C Etteri S Maggi A. The endogenous estrogen status regulates microglia reactivity in animal models of neuroinflammation. Endocrinology (2006) 147(5):2263–72. 10.1210/en.2005-1330 16469811

[70] Drew PD Chavis JA. Female sex steroids: effects upon microglial cell activation. J Neuroimmunol (2000) 111(1–2):77–85. 10.1016/S0165-5728(00)00386-6 11063824

[71] Vegeto E Bonincontro C Pollio G Sala A Viappiani S Nardi F Estrogen prevents the lipopolysaccharide-induced inflammatory response in microglia. J Neurosci (2001) 21(6):1809–18. 11245665 10.1523/JNEUROSCI.21-06-01809.2001 PMC6762610

[72] Tenenbaum M Azab AN Kaplanski J. Effects of estrogen against LPS-induced inflammation and toxicity in primary rat glial and neuronal cultures. J Endotoxin Res (2007) 13(3):158–66. 10.1177/0968051907080428 17621558

[73] Baker AE Brautigam VM Watters JJ. Estrogen modulates microglial inflammatory mediator production via interactions with estrogen receptor beta. Endocrinology (2004) 145(11):5021–32. 10.1210/en.2004-0619 15256495

[74] Loane DJ Kumar A. Microglia in the TBI brain: the good, the bad, and the dysregulated. Exp Neurol (2016) 275(Pt 3):316–27. 10.1016/j.expneurol.2015.08.018 26342753 PMC4689601

[75] Garcia-Estrada J Del Rio JA Luquin S Soriano E Garcia-Segura LM. Gonadal hormones down-regulate reactive gliosis and astrocyte proliferation after a penetrating brain injury. Brain Res (1993) 628(1–2):271–8. 10.1016/0006-8993(93)90964-O 8313156

[76] Barreto G Santos-Galindo M Diz-Chaves Y Pernia O Carrero P Azcoitia I Selective estrogen receptor modulators decrease reactive astrogliosis in the injured brain: effects of aging and prolonged depletion of ovarian hormones. Endocrinology (2009) 150(11):5010–5. 10.1210/en.2009-0352 19797123

[77] Ciriza I Carrero P Azcoitia I Lundeen SG Garcia-Segura LM. Selective estrogen receptor modulators protect hippocampal neurons from kainic acid excitotoxicity: differences with the effect of estradiol. J Neurobiol (2004) 61(2):209–21. 10.1002/neu.20043 15389604

[78] Tripanichkul W Sripanichkulchai K Finkelstein DI. Estrogen down-regulates glial activation in male mice following 1-methyl-4-phenyl-1,2,3,6-tetrahydropyridine intoxication. Brain Res (2006) 1084(1):28–37. 10.1016/j.brainres.2006.02.029 16564034

[79] Valles SL Dolz-Gaiton P Gambini J Borras C Lloret A Pallardo FV Estradiol or genistein prevent Alzheimer’s disease-associated inflammation correlating with an increase PPAR gamma expression in cultured astrocytes. Brain Res (2010) 1312:138–44. 10.1016/j.brainres.2009.11.044 19948157

[80] Kireev RA Vara E Vina J Tresguerres JA. Melatonin and oestrogen treatments were able to improve neuroinflammation and apoptotic processes in dentate gyrus of old ovariectomized female rats. Age (Dordr) (2014) 36(5):9707. 10.1007/s11357-014-9707-3 25135305 PMC4453938

[81] Cerciat M Unkila M Garcia-Segura LM Arevalo MA. Selective estrogen receptor modulators decrease the production of interleukin-6 and interferon-gamma-inducible protein-10 by astrocytes exposed to inflammatory challenge in vitro. Glia (2010) 58(1):93–102. 10.1002/glia.20904 19533603

[82] Spence RD Hamby ME Umeda E Itoh N Du S Wisdom AJ Neuroprotection mediated through estrogen receptor-alpha in astrocytes. Proc Natl Acad Sci U S A (2011) 108(21):8867–72. 10.1073/pnas.1103833108 21555578 PMC3102368

[83] Guo J Duckles SP Weiss JH Li X Krause DN 17Beta-estradiol prevents cell death and mitochondrial dysfunction by an estrogen receptor-dependent mechanism in astrocytes after oxygen-glucose deprivation/reperfusion. Free Radic Biol Med (2012) 52(11–12):2151–60. 10.1016/j.freeradbiomed.2012.03.005 22554613 PMC3377773

[84] De Marinis E Acaz-Fonseca E Arevalo MA Ascenzi P Fiocchetti M Marino M 17Beta-oestradiol anti-inflammatory effects in primary astrocytes require oestrogen receptor beta-mediated neuroglobin up-regulation. J Neuroendocrinol (2013) 25(3):260–70. 10.1111/jne.12007 23190172

[85] Lee E Sidoryk-Wegrzynowicz M Farina M Rocha JB Aschner M. Estrogen attenuates manganese-induced glutamate transporter impairment in rat primary astrocytes. Neurotox Res (2013) 23(2):124–30. 10.1007/s12640-012-9347-2 22878846 PMC3681521

[86] Sarkaki AR Khaksari Haddad M Soltani Z Shahrokhi N Mahmoodi M. Time- and dose-dependent neuroprotective effects of sex steroid hormones on inflammatory cytokines after a traumatic brain injury. J Neurotrauma (2013) 30(1):47–54. 10.1089/neu.2010.1686 21851230

[87] Zhang QG Wang R Tang H Dong Y Chan A Sareddy GR Brain-derived estrogen exerts anti-inflammatory and neuroprotective actions in the rat hippocampus. Mol Cell Endocrinol (2014) 389(1–2):84–91. 10.1016/j.mce.2013.12.019 24508637 PMC4040313

[88] Marriott LK Hauss-Wegrzyniak B Benton RS Vraniak PD Wenk GL. Long-term estrogen therapy worsens the behavioral and neuropathological consequences of chronic brain inflammation. Behav Neurosci (2002) 116(5):902–11. 10.1037/0735-7044.116.5.902 12369809

[89] Nordell VL Scarborough MM Buchanan AK Sohrabji F. Differential effects of estrogen in the injured forebrain of young adult and reproductive senescent animals. Neurobiol Aging (2003) 24(5):733–43. 10.1016/S0197-4580(02)00193-8 12885581

[90] Cunningham MA Wirth JR Freeman LR Boger HA Granholm AC Gilkeson GS. Estrogen receptor alpha deficiency protects against development of cognitive impairment in murine lupus. J Neuroinflammation (2014) 11:171. 10.1186/s12974-014-0171-x 25510908 PMC4272530

[91] Ghisletti S Meda C Maggi A Vegeto E. 17Beta-estradiol inhibits inflammatory gene expression by controlling NF-kappaB intracellular localization. Mol Cell Biol (2005) 25(8):2957–68. 10.1128/MCB.25.8.2957-2968.2005 15798185 PMC1069609

[92] Sarvari M Hrabovszky E Kallo I Solymosi N Toth K Liko I Estrogens regulate neuroinflammatory genes via estrogen receptors alpha and beta in the frontal cortex of middle-aged female rats. J Neuroinflammation (2011) 8:82 10.1186/1742-2094-8-82 21774811 PMC3161870

[93] Bilbo SD Tsang V. Enduring consequences of maternal obesity for brain inflammation and behavior of offspring. FASEB J (2010) 24(6):2104–15. 10.1096/fj.09-144014 20124437

[94] Sasaki A de Vega WC St-Cyr S Pan P McGowan PO. Perinatal high fat diet alters glucocorticoid signaling and anxiety behavior in adulthood. Neuroscience (2013) 240:1–12. 10.1016/j.neuroscience.2013.02.044 23454542

[95] Bellisario V Panetta P Balsevich G Baumann V Noble J Raggi C Maternal high-fat diet acts as a stressor increasing maternal glucocorticoids’ signaling to the fetus and disrupting maternal behavior and brain activation in C57BL/6J mice. Psychoneuroendocrinology (2015) 60:138–50. 10.1016/j.psyneuen.2015.06.012 26143538

[96] Sasaki A de Vega W Sivanathan S St-Cyr S McGowan PO. Maternal high-fat diet alters anxiety behavior and glucocorticoid signaling in adolescent offspring. Neuroscience (2014) 272:92–101. 10.1016/j.neuroscience.2014.04.012 24791714

[97] Tannenbaum BM Brindley DN Tannenbaum GS Dallman MF McArthur MD Meaney MJ. High-fat feeding alters both basal and stress-induced hypothalamic-pituitary-adrenal activity in the rat. Am J Physiol (1997) 273(6 Pt 1):E1168–77. 9435533 10.1152/ajpendo.1997.273.6.E1168

[98] Shin AC MohanKumar SM Sirivelu MP Claycombe KJ Haywood JR Fink GD Chronic exposure to a high-fat diet affects stress axis function differentially in diet-induced obese and diet-resistant rats. Int J Obes (Lond) (2010) 34(7):1218–26. 10.1038/ijo.2010.34 20212497 PMC2892636

[99] Bray GA Lovejoy JC Smith SR DeLany JP Lefevre M Hwang D The influence of different fats and fatty acids on obesity, insulin resistance and inflammation. J Nutr (2002) 132(9):2488–91. 12221198 10.1093/jn/132.9.2488

[100] Thaler JP Schwartz MW. Minireview: inflammation and obesity pathogenesis: the hypothalamus heats up. Endocrinology (2010) 151(9):4109–15. 10.1210/en.2010-0336 20573720 PMC2940486

[101] Sivanathan S Thavartnam K Arif S Elegino T McGowan PO. Chronic high fat feeding increases anxiety-like behaviour and reduces transcript abundance of glucocorticoid signalling genes in the hippocampus of female rats. Behav Brain Res (2015) 286:265–70. 10.1016/j.bbr.2015.02.036 25721737

[102] Herman JP Ostrander MM Mueller NK Figueiredo H. Limbic system mechanisms of stress regulation: hypothalamo-pituitary-adrenocortical axis. Prog Neuropsychopharmacol Biol Psychiatry (2005) 29(8):1201–13. 10.1016/j.pnpbp.2005.08.006 16271821

[103] Sapolsky RM Krey LC McEwen BS The neuroendocrinology of stress and aging: the glucocorticoid cascade hypothesis. Endocr Rev (1986) 7(3):284–301. 10.1210/edrv-7-3-284 3527687

[104] Issa AM Rowe W Gauthier S Meaney MJ. Hypothalamic-pituitary-adrenal activity in aged, cognitively impaired and cognitively unimpaired rats. J Neurosci (1990) 10(10):3247–54. 2170594 10.1523/JNEUROSCI.10-10-03247.1990 PMC6570181

[105] Tarcic N Ovadia H Weiss DW Weidenfeld J. Restraint stress-induced thymic involution and cell apoptosis are dependent on endogenous glucocorticoids. J Neuroimmunol (1998) 82(1):40–6. 10.1016/S0165-5728(97)00186-0 9526844

[106] De Bosscher K Vanden Berghe W Haegeman G. The interplay between the glucocorticoid receptor and nuclear factor-kappaB or activator protein-1: molecular mechanisms for gene repression. Endocr Rev (2003) 24(4):488–522. 10.1210/er.2002-0006 12920152

[107] Wurtman RJ Axelrod J. Control of enzymatic synthesis of adrenaline in adrenal medulla by adrenal cortical steroids. J Biol Chem (1966) 241(10):2301–5. 4287854

[108] Lindqvist A Mohapel P Bouter B Frielingsdorf H Pizzo D Brundin P High-fat diet impairs hippocampal neurogenesis in male rats. Eur J Neurol (2006) 13(12):1385–8. 10.1111/j.1468-1331.2006.01500.x 17116226

[109] Molteni R Barnard RJ Ying Z Roberts CK Gomez-Pinilla F. A high-fat, refined sugar diet reduces hippocampal brain-derived neurotrophic factor, neuronal plasticity, and learning. Neuroscience (2002) 112(4):803–14. 10.1016/S0306-4522(02)00123-9 12088740

[110] Wu A Molteni R Ying Z Gomez-Pinilla F. A saturated-fat diet aggravates the outcome of traumatic brain injury on hippocampal plasticity and cognitive function by reducing brain-derived neurotrophic factor. Neuroscience (2003) 119(2):365–75. 10.1016/S0306-4522(03)00154-4 12770552

[111] Granholm AC Bimonte-Nelson HA Moore AB Nelson ME Freeman LR Sambamurti K. Effects of a saturated fat and high cholesterol diet on memory and hippocampal morphology in the middle-aged rat. J Alzheimers Dis (2008) 14(2):133–45. 18560126 10.3233/jad-2008-14202 PMC2670571

[112] Ambrogini P Orsini L Mancini C Ferri P Barbanti I Cuppini R. Persistently high corticosterone levels but not normal circadian fluctuations of the hormone affect cell proliferation in the adult rat dentate gyrus. Neuroendocrinology (2002) 76(6):366–72. 10.1159/000067581 12566944

[113] Heine VM Maslam S Joels M Lucassen PJ. Prominent decline of newborn cell proliferation, differentiation, and apoptosis in the aging dentate gyrus, in absence of an age-related hypothalamus-pituitary-adrenal axis activation. Neurobiol Aging (2004) 25(3):361–75. 10.1016/S0197-4580(03)00090-3 15123342

[114] Farr SA Banks WA Morley JE. Effects of leptin on memory processing. Peptides (2006) 27(6):1420–5. 10.1016/j.peptides.2005.10.006 16293343

[115] Arnold SE Lucki I Brookshire BR Carlson GC Browne CA Kazi H High fat diet produces brain insulin resistance, synaptodendritic abnormalities and altered behavior in mice. Neurobiol Dis (2014) 67:79–87. 10.1016/j.nbd.2014.03.011 24686304 PMC4083060

[116] Molteni R Wu A Vaynman S Ying Z Barnard RJ Gomez-Pinilla F. Exercise reverses the harmful effects of consumption of a high-fat diet on synaptic and behavioral plasticity associated to the action of brain-derived neurotrophic factor. Neuroscience (2004) 123(2):429–40. 10.1016/j.neuroscience.2003.09.020 14698750

[117] Boitard C Cavaroc A Sauvant J Aubert A Castanon N Laye S Impairment of hippocampal-dependent memory induced by juvenile high-fat diet intake is associated with enhanced hippocampal inflammation in rats. Brain Behav Immun (2014) 40:9–17. 10.1016/j.bbi.2014.03.005 24662056

[118] Liu Y Fu X Lan N Li S Zhang J Wang S Luteolin protects against high fat diet-induced cognitive deficits in obesity mice. Behav Brain Res (2014) 267:178–88. 10.1016/j.bbr.2014.02.040 24667364

[119] Kanoski SE Zhang Y Zheng W Davidson TL. The effects of a high-energy diet on hippocampal function and blood-brain barrier integrity in the rat. J Alzheimers Dis (2010) 21(1):207–19. 10.3233/JAD-2010-091414 20413889 PMC4975946

[120] Davidson TL Monnot A Neal AU Martin AA Horton JJ Zheng W. The effects of a high-energy diet on hippocampal-dependent discrimination performance and blood-brain barrier integrity differ for diet-induced obese and diet-resistant rats. Physiol Behav (2012) 107(1):26–33. 10.1016/j.physbeh.2012.05.015 22634281 PMC3409296

[121] Davidson TL Hargrave SL Swithers SE Sample CH Fu X Kinzig KP Inter-relationships among diet, obesity and hippocampal-dependent cognitive function. Neuroscience (2013) 253:110–22. 10.1016/j.neuroscience.2013.08.044 23999121 PMC3934926

[122] Mulder M Blokland A van den Berg DJ Schulten H Bakker AH Terwel D Apolipoprotein E protects against neuropathology induced by a high-fat diet and maintains the integrity of the blood-brain barrier during aging. Lab Invest (2001) 81(7):953–60. 10.1038/labinvest.3780307 11454984

[123] Hsu TM Kanoski SE. Blood-brain barrier disruption: mechanistic links between Western diet consumption and dementia. Front Aging Neurosci (2014) 6:88. 10.3389/fnagi.2014.00088 24847262 PMC4023063

[124] Thomas HV Reeves GK Key TJ. Endogenous estrogen and postmenopausal breast cancer: a quantitative review. Cancer Causes Control (1997) 8(6):922–8. 10.1023/A:1018476631561 9427435

[125] Prentice R Thompson D Clifford C Gorbach S Goldin B Byar D. Dietary fat reduction and plasma estradiol concentration in healthy postmenopausal women. The Women’s Health Trial Study Group. J Natl Cancer Inst (1990) 82(2):129–34. 10.1093/jnci/82.2.129 2294222

[126] Heber D Ashley JM Leaf DA Barnard RJ. Reduction of serum estradiol in postmenopausal women given free access to low-fat high-carbohydrate diet. Nutrition (1991) 7(2):137–9. 1666318

[127] Goldin BR Adlercreutz H Gorbach SL Woods MN Dwyer JT Conlon T The relationship between estrogen levels and diets of Caucasian American and Oriental immigrant women. Am J Clin Nutr (1986) 44(6):945–53. 3024478 10.1093/ajcn/44.6.945

[128] Rose DP Boyar AP Cohen C Strong LE. Effect of a low-fat diet on hormone levels in women with cystic breast disease. I. Serum steroids and gonadotropins. J Natl Cancer Inst (1987) 78(4):623–6. 3104646

[129] Woods MN Gorbach SL Longcope C Goldin BR Dwyer JT Morrill-LaBrode A. Low-fat, high-fiber diet and serum estrone sulfate in premenopausal women. Am J Clin Nutr (1989) 49(6):1179–83. 2543203 10.1093/ajcn/49.6.1179

[130] Bagga D Ashley JM Geffrey SP Wang HJ Barnard RJ Korenman S Effects of a very low fat, high fiber diet on serum hormones and menstrual function. Implications for breast cancer prevention. Cancer (1995) 76(12):2491–6. 10.1002/1097-0142(19951215)76:12<2491::AID-CNCR2820761213>3.0.CO;2-R 8625075

[131] James MJ Gibson RA Cleland LG. Dietary polyunsaturated fatty acids and inflammatory mediator production. Am J Clin Nutr (2000) 71(1 Suppl):343S–8S. 10617994 10.1093/ajcn/71.1.343s

[132] Kelley DS. Modulation of human immune and inflammatory responses by dietary fatty acids. Nutrition (2001) 17(7–8):669–73. 10.1016/S0899-9007(01)00576-7 11448594

[133] Noble LS Takayama K Zeitoun KM Putman JM Johns DA Hinshelwood MM Prostaglandin E2 stimulates aromatase expression in endometriosis-derived stromal cells. J Clin Endocrinol Metab (1997) 82(2):600–6. 10.1210/jcem.82.2.3783 9024261

[134] Young LR Kurzer MS Thomas W Redmon JB Raatz SK. Effect of dietary fat and omega-3 fatty acids on urinary eicosanoids and sex hormone concentrations in postmenopausal women: a randomized controlled feeding trial. Nutr Cancer (2011) 63(6):930–9. 10.1080/01635581.2011.589957 21745038

[135] Scott E Zhang QG Wang R Vadlamudi R Brann D. Estrogen neuroprotection and the critical period hypothesis. Front Neuroendocrinol (2012) 33(1):85–104. 10.1016/j.yfrne.2011.10.001 22079780 PMC3288697

[136] Fan Y Liu Y Xue K Gu G Fan W Xu Y Diet-induced obesity in male C57BL/6 mice decreases fertility as a consequence of disrupted blood-testis barrier. PLoS One (2015) 10(4):e0120775. 10.1371/journal.pone.0120775 25886196 PMC4401673

[137] Yan WJ Mu Y Yu N Yi TL Zhang Y Pang XL Protective effects of metformin on reproductive function in obese male rats induced by high-fat diet. J Assist Reprod Genet (2015) 32(7):1097–104. 10.1007/s10815-015-0506-2 26081124 PMC4531858

[138] French SA Jeffery RW. Consequences of dieting to lose weight: effects on physical and mental health. Health Psychol (1994) 13(3):195–212. 10.1037/0278-6133.13.3.195 8055855

[139] Weissman C. The metabolic response to stress: an overview and update. Anesthesiology (1990) 73(2):308–27. 10.1097/00000542-199008000-00020 2200312

[140] Mattson MP. Hormesis defined. Ageing Res Rev (2008) 7(1):1–7. 10.1016/j.arr.2007.08.007 18162444 PMC2248601

[141] Munck A Guyre PM Holbrook NJ. Physiological functions of glucocorticoids in stress and their relation to pharmacological actions. Endocr Rev (1984) 5(1):25–44. 10.1210/edrv-5-1-25 6368214

[142] Yu BP Chung HY. Stress resistance by caloric restriction for longevity. Ann N Y Acad Sci (2001) 928:39–47. 10.1111/j.1749-6632.2001.tb05633.x 11795526

[143] Mattson MP Chan SL Duan W. Modification of brain aging and neurodegenerative disorders by genes, diet, and behavior. Physiol Rev (2002) 82(3):637–72. 10.1152/physrev.00004.2002 12087131

[144] Mattson MP Duan W Wan R Guo Z. Prophylactic activation of neuroprotective stress response pathways by dietary and behavioral manipulations. NeuroRx (2004) 1(1):111–6. 10.1602/neurorx.1.1.111 15717011 PMC534916

[145] Dhurandhar EJ Allison DB van Groen T Kadish I. Hunger in the absence of caloric restriction improves cognition and attenuates Alzheimer’s disease pathology in a mouse model. PLoS One (2013) 8(4):e60437. 10.1371/journal.pone.0060437 23565247 PMC3614512

[146] Nelson JF Karelus K Bergman MD Felicio LS. Neuroendocrine involvement in aging: evidence from studies of reproductive aging and caloric restriction. Neurobiol Aging (1995) 16(5):837–43. 10.1016/0197-4580(95)00072-M 8532119

[147] Armario A Montero JL Jolin T. Chronic food restriction and the circadian rhythms of pituitary-adrenal hormones, growth hormone and thyroid-stimulating hormone. Ann Nutr Metab (1987) 31(2):81–7. 10.1159/000177254 3035995

[148] Han ES Levin N Bengani N Roberts JL Suh Y Karelus K Hyperadrenocorticism and food restriction-induced life extension in the rat: evidence for divergent regulation of pituitary proopiomelanocortin RNA and adrenocorticotropic hormone biosynthesis. J Gerontol A Biol Sci Med Sci (1995) 50(5):B288–94. 10.1093/gerona/50A.5.B288 7545529

[149] Klebanov S Diais S Stavinoha WB Suh Y Nelson JF. Hyperadrenocorticism, attenuated inflammation, and the life-prolonging action of food restriction in mice. J Gerontol A Biol Sci Med Sci (1995) 50(2):B78–82. 10.1093/gerona/50A.2.B78 7874583

[150] Heiderstadt KM McLaughlin RM Wright DC Walker SE Gomez-Sanchez CE. The effect of chronic food and water restriction on open-field behaviour and serum corticosterone levels in rats. Lab Anim (2000) 34(1):20–8. 10.1258/002367700780578028 10759363

[151] Han ES Evans TR Shu JH Lee S Nelson JF. Food restriction enhances endogenous and corticotropin-induced plasma elevations of free but not total corticosterone throughout life in rats. J Gerontol A Biol Sci Med Sci (2001) 56(9):B391–7. 10.1093/gerona/56.9.B391 11524440

[152] Sabatino F Masoro EJ McMahan CA Kuhn RW. Assessment of the role of the glucocorticoid system in aging processes and in the action of food restriction. J Gerontol (1991) 46(5):B171–9. 10.1093/geronj/46.5.B171 1890278

[153] Harper JM Leathers CW Austad SN. Does caloric restriction extend life in wild mice? Aging Cell (2006) 5(6):441–9. 10.1111/j.1474-9726.2006.00236.x 17054664 PMC2923404

[154] Tomiyama AJ Mann T Vinas D Hunger JM Dejager J Taylor SE. Low calorie dieting increases cortisol. Psychosom Med (2010) 72(4):357–64. 10.1097/PSY.0b013e3181d9523c 20368473 PMC2895000

[155] Villanueva AL Schlosser C Hopper B Liu JH Hoffman DI Rebar RW. Increased cortisol production in women runners. J Clin Endocrinol Metab (1986) 63(1):133–6. 10.1210/jcem-63-1-133 3011836

[156] Casper RC Chatterton RT Jr Davis JM. Alterations in serum cortisol and its binding characteristics in anorexia nervosa. J Clin Endocrinol Metab (1979) 49(3):406–11. 10.1210/jcem-49-3-406 468975

[157] Walford RL Mock D Verdery R MacCallum T. Calorie restriction in biosphere 2: alterations in physiologic, hematologic, hormonal, and biochemical parameters in humans restricted for a 2-year period. J Gerontol A Biol Sci Med Sci (2002) 57(6):B211–24. 10.1093/gerona/57.6.B211 12023257

[158] Grayson BE Hakala-Finch AP Kekulawala M Laub H Egan AE Ressler IB Weight loss by calorie restriction versus bariatric surgery differentially regulates the hypothalamo-pituitary-adrenocortical axis in male rats. Stress (2014) 17(6):484–93. 10.3109/10253890.2014.967677 25238021 PMC4415587

[159] Morimoto Y Arisue K Yamamura Y Relationship between circadian rhythm of food intake and that of plasma corticosterone and effect of food restriction on circadian adrenocortical rhythm in the rat. Neuroendocrinology (1977) 23(4):212–22. 10.1159/000122669 909626

[160] Yaktine AL Vaughn R Blackwood D Duysen E Birt DF. Dietary energy restriction in the SENCAR mouse: elevation of glucocorticoid hormone levels but no change in distribution of glucocorticoid receptor in epidermal cells. Mol Carcinog (1998) 21(1):62–9. 10.1002/(SICI)1098-2744(199801)21:1<62::AID-MC8>3.3.CO;2-8 9473772

[161] al-Hadramy MS Zawawi TH Abdelwahab SM. Altered cortisol levels in relation to Ramadan. Eur J Clin Nutr (1988) 42(4):359–62. 3396527

[162] Sliman NA Ajlouni KS Faisal K Effect of fasting on some blood hormones in healthy Muslim males. Mutah J Res Stud (1993) 8:91–109.

[163] Ben Salem L B’Chir S Bchir F Bouguerra R Ben Slama C. [Circadian rhythm of cortisol and its responsiveness to ACTH during Ramadan]. Ann Endocrinol (2002) 63(6 Pt 1):497–501.AE-12-2002-63-6-0003-4266-101019-ART8 12527850

[164] El-Migdadi F El-Akawi Z Abudheese R Bashir N. Plasma levels of adrenocorticotropic hormone and cortisol in people living in an environment below sea level (Jordan Valley) during fasting in the month of Ramadan. Horm Res (2002) 58(6):279–82. 10.1159/000066446 12446991

[165] Faris MA Kacimi S Al-Kurd RA Fararjeh MA Bustanji YK Mohammad MK Intermittent fasting during Ramadan attenuates proinflammatory cytokines and immune cells in healthy subjects. Nutr Res (2012) 32(12):947–55. 10.1016/j.nutres.2012.06.021 23244540

[166] Akrami Mohajeri F Ahmadi Z Hassanshahi G Akrami Mohajeri E Ravari A Ghalebi SR. Dose Ramadan fasting affects inflammatory responses: evidences for modulatory roles of this unique nutritional status via chemokine network. Iran J Basic Med Sci (2013) 16(12):1217–22. 24570826 PMC3933797

[167] Tam CS Frost EA Xie W Rood J Ravussin E Redman LM No effect of caloric restriction on salivary cortisol levels in overweight men and women. Metabolism (2014) 63(2):194–8. 10.1016/j.metabol.2013.10.007 24268369 PMC3946997

[168] Johnstone AM Faber P Andrew R Gibney ER Elia M Lobley G Influence of short-term dietary weight loss on cortisol secretion and metabolism in obese men. Eur J Endocrinol (2004) 150(2):185–94. 10.1530/eje.0.1500185 14763916

[169] Sticker LS Thompson DL Jr Fernandez JM Bunting LD DePew CL. Dietary protein and(or) energy restriction in mares: plasma growth hormone, IGF-I, prolactin, cortisol, and thyroid hormone responses to feeding, glucose, and epinephrine. J Anim Sci (1995) 73(5):1424–32. 7665373 10.2527/1995.7351424x

[170] Glade MJ Gupta S Reimers TJ. Hormonal responses to high and low planes of nutrition in weanling thoroughbreds. J Anim Sci (1984) 59(3):658–65. 6386779 10.2527/jas1984.593658x

[171] Smith-Swintosky VL Pettigrew LC Sapolsky RM Phares C Craddock SD Brooke SM Metyrapone, an inhibitor of glucocorticoid production, reduces brain injury induced by focal and global ischemia and seizures. J Cereb Blood Flow Metab (1996) 16(4):585–98. 10.1097/00004647-199607000-00008 8964797

[172] Aly KB Pipkin JL Hinson WG Feuers RJ Duffy PH Lyn-Cook L Chronic caloric restriction induces stress proteins in the hypothalamus of rats. Mech Ageing Dev (1994) 76(1):11–23. 10.1016/0047-6374(94)90002-7 7845058

[173] Yu ZF Mattson MP. Dietary restriction and 2-deoxyglucose administration reduce focal ischemic brain damage and improve behavioral outcome: evidence for a preconditioning mechanism. J Neurosci Res (1999) 57(6):830–9. 10.1002/(SICI)1097-4547(19990915)57:6<830::AID-JNR8>3.3.CO;2-U 10467254

[174] Zatsepina OG Evgen’ev MB Liashchko VN. [Changes in the transcription activity of c-myc genes and heat shock proteins (HSP 70) after incubation of mouse plasmacytoma cells with dexamethasone]. Mol Biol (1990) 24(2):391–5. 2362589

[175] Lee J Herman JP Mattson MP. Dietary restriction selectively decreases glucocorticoid receptor expression in the hippocampus and cerebral cortex of rats. Exp Neurol (2000) 166(2):435–41. 10.1006/exnr.2000.7512 11085908

[176] Vasconcelos AR Yshii LM Viel TA Buck HS Mattson MP Scavone C Intermittent fasting attenuates lipopolysaccharide-induced neuroinflammation and memory impairment. J Neuroinflammation (2014) 11:85. 10.1186/1742-2094-11-85 24886300 PMC4041059

[177] MacDonald L Radler M Paolini AG Kent S. Calorie restriction attenuates LPS-induced sickness behavior and shifts hypothalamic signaling pathways to an anti-inflammatory bias. Am J Physiol Regul Integr Comp Physiol (2011) 301(1):R172–84. 10.1152/ajpregu.00057.2011 21525175

[178] MacDonald L Hazi A Paolini AG Kent S. Calorie restriction dose-dependently abates lipopolysaccharide-induced fever, sickness behavior, and circulating interleukin-6 while increasing corticosterone. Brain Behav Immun (2014) 40:18–26. 10.1016/j.bbi.2014.01.005 24440143

[179] Matsuzaki J Kuwamura M Yamaji R Inui H Nakano Y. Inflammatory responses to lipopolysaccharide are suppressed in 40% energy-restricted mice. J Nutr (2001) 131(8):2139–44. 11481408 10.1093/jn/131.8.2139

[180] Longo VD Fontana L. Calorie restriction and cancer prevention: metabolic and molecular mechanisms. Trends Pharmacol Sci (2010) 31(2):89–98. 10.1016/j.tips.2009.11.004 20097433 PMC2829867

[181] Trainin N Adrenal imbalance in mouse skin carcinogenesis. Cancer Res (1963) 23:415–9. 13993953

[182] Belman S Troll W The inhibition of croton oil-promoted mouse skin tumorigenesis by steroid hormones. Cancer Res (1972) 32(3):450–4. 5061300

[183] Mitev Y Almeida OF Patchev V. Pituitary-adrenal function and hypothalamic beta-endorphin release in vitro following food deprivation. Brain Res Bull (1993) 30(1–2):7–10. 10.1016/0361-9230(93)90033-8 8420637

[184] Zhu Z Jiang W Thompson HJ. An experimental paradigm for studying the cellular and molecular mechanisms of cancer inhibition by energy restriction. Mol Carcinog (2002) 35(2):51–6. 10.1002/mc.10073 12325034

[185] Zhu Z Jiang W Thompson HJ. Mechanisms by which energy restriction inhibits rat mammary carcinogenesis: in vivo effects of corticosterone on cell cycle machinery in mammary carcinomas. Carcinogenesis (2003) 24(7):1225–31. 10.1093/carcin/bgg077 12807724

[186] Pashko LL Schwartz AG. Reversal of food restriction-induced inhibition of mouse skin tumor promotion by adrenalectomy. Carcinogenesis (1992) 13(10):1925–8. 10.1093/carcin/13.10.1925 1423856

[187] Pashko LL Schwartz AG. Inhibition of 7,12-dimethylbenz[a]anthracene-induced lung tumorigenesis in A/J mice by food restriction is reversed by adrenalectomy. Carcinogenesis (1996) 17(2):209–12. 10.1093/carcin/17.2.209 8625440

[188] Stewart JW Koehler K Jackson W Hawley J Wang W Au A Prevention of mouse skin tumor promotion by dietary energy restriction requires an intact adrenal gland and glucocorticoid supplementation restores inhibition. Carcinogenesis (2005) 26(6):1077–84. 10.1093/carcin/bgi051 15746164

[189] Akiyama H Barger S Barnum S Bradt B Bauer J Cole GM Inflammation and Alzheimer’s disease. Neurobiol Aging (2000) 21(3):383–421. 10.1016/S0197-4580(00)00124-X 10858586 PMC3887148

[190] Fito M Guxens M Corella D Saez G Estruch R de la Torre R Effect of a traditional Mediterranean diet on lipoprotein oxidation: a randomized controlled trial. Arch Intern Med (2007) 167(11):1195–203. 10.1001/archinte.167.11.1195 17563030

[191] Floyd RA. Neuroinflammatory processes are important in neurodegenerative diseases: an hypothesis to explain the increased formation of reactive oxygen and nitrogen species as major factors involved in neurodegenerative disease development. Free Radic Biol Med (1999) 26(9–10):1346–55. 10.1016/S0891-5849(98)00293-7 10381209

[192] Teunissen CE Lutjohann D von Bergmann K Verhey F Vreeling F Wauters A Combination of serum markers related to several mechanisms in Alzheimer’s disease. Neurobiol Aging (2003) 24(7):893–902. 10.1016/S0197-4580(03)00005-8 12928047

[193] DeLegge MH Smoke A. Neurodegeneration and inflammation. Nutr Clin Pract (2008) 23(1):35–41. 10.1177/011542650802300135 18203962

[194] Bruce-Keller AJ Umberger G McFall R Mattson MP. Food restriction reduces brain damage and improves behavioral outcome following excitotoxic and metabolic insults. Ann Neurol (1999) 45(1):8–15. 10.1002/1531-8249(199901)45:1<8::AID-ART4>3.3.CO;2-M 9894871

[195] Arumugam TV Phillips TM Cheng A Morrell CH Mattson MP Wan R. Age and energy intake interact to modify cell stress pathways and stroke outcome. Ann Neurol (2010) 67(1):41–52. 10.1002/ana.21798 20186857 PMC2844782

[196] Fann DY Santro T Manzanero S Widiapradja A Cheng YL Lee SY Intermittent fasting attenuates inflammasome activity in ischemic stroke. Exp Neurol (2014) 257:114–9. 10.1016/j.expneurol.2014.04.017 24805069

[197] Hatzinger M Z’Brun A Hemmeter U Seifritz E Baumann F Holsboer-Trachsler E Hypothalamic-pituitary-adrenal system function in patients with Alzheimer’s disease. Neurobiol Aging (1995) 16(2):205–9. 10.1016/0197-4580(94)00159-6 7777138

[198] Goodman Y Bruce AJ Cheng B Mattson MP. Estrogens attenuate and corticosterone exacerbates excitotoxicity, oxidative injury, and amyloid beta-peptide toxicity in hippocampal neurons. J Neurochem (1996) 66(5):1836–44. 10.1046/j.1471-4159.1996.66051836.x 8780008

[199] Pedersen WA Culmsee C Ziegler D Herman JP Mattson MP. Aberrant stress response associated with severe hypoglycemia in a transgenic mouse model of Alzheimer’s disease. J Mol Neurosci (1999) 13(1–2):159–65. 10.1385/JMN:13:1-2:159 10691302

[200] Peck MD Babcock GF Alexander JW. The role of protein and calorie restriction in outcome from *Salmonella* infection in mice. JPEN J Parenter Enteral Nutr (1992) 16(6):561–5. 10.1177/0148607192016006561 1494214

[201] Dong W Selgrade MK Gilmour IM Lange RW Park P Luster MI Altered alveolar macrophage function in calorie-restricted rats. Am J Respir Cell Mol Biol (1998) 19(3):462–9. 10.1165/ajrcmb.19.3.3114 9730874

[202] Mascarucci P Taub D Saccani S Paloma MA Dawson H Roth GS Cytokine responses in young and old rhesus monkeys: effect of caloric restriction. J Interferon Cytokine Res (2002) 22(5):565–71. 10.1089/10799900252982043 12060495

[203] Jolly CA. Dietary restriction and immune function. J Nutr (2004) 134(8):1853–6. 15284365 10.1093/jn/134.8.1853

[204] Nayak BN Friel JK Rempel CB Jones PJ. Energy-restricted diets result in higher numbers of CD4+, CD8+, immunoglobulins (A, M, and G), and CD45RA cells in spleen and CD4+, immunoglobulin A, and CD45RA cells in colonic lamina propria of rats. Nutr Res (2009) 29(7):487–93. 10.1016/j.nutres.2009.06.010 19700036

[205] Patel NV Finch CE. The glucocorticoid paradox of caloric restriction in slowing brain aging. Neurobiol Aging (2002) 23(5):707–17. 10.1016/S0197-4580(02)00017-9 12392776

[206] Harvie MN Pegington M Mattson MP Frystyk J Dillon B Evans G The effects of intermittent or continuous energy restriction on weight loss and metabolic disease risk markers: a randomized trial in young overweight women. Int J Obes (Lond) (2011) 35(5):714–27. 10.1038/ijo.2010.171 20921964 PMC3017674

